# EVOLUTION OF BOWEL FUNCTION AND QUALITY OF LIFE AFTER SPINAL CORD INJURY: A LONGITUDINAL REGISTRY AND SURVEY STUDY

**DOI:** 10.2340/jrm.v58.44175

**Published:** 2026-01-15

**Authors:** Charlotta JOSEFSON, Katharina Stibrant SUNNERHAGEN

**Affiliations:** 1 Sahlgrenska Academy, University of Gothenburg, Gothenburg; 2 Sahlgrenska University Hospital, Gothenburg, Sweden

**Keywords:** spinal cord injuries, neurogenic bowel, quality of life

## Abstract

**Objective:**

To assess changes in bowel function and quality of life over time in adults with spinal cord injury.

**Design:**

Retrospective cohort study linking clinical registry data with patient-reported outcomes.

**Subjects:**

236 adults with spinal cord injury in Sweden; 157 had both baseline and follow-up data.

**Methods:**

Data from the Swedish National Quality Registry for Rehabilitation Medicine were merged with responses from the 2024 Swedish International Spinal Cord Injury Community Survey. Bowel dysfunction was assessed using the Spinal Cord Injury Secondary Conditions Scale, Constipation Scoring System, and St. Mark’s Incontinence Score. Quality of life was measured using the 3L EQ-5D questionnaire at baseline and the 5L version at follow-up, converted to a common scale. Group differences were analysed using χ^2^ and Mann–Whitney *U* tests. Logistic regression identified predictors of bowel outcomes; linear regression assessed factors associated with quality-of-life scores.

**Results:**

At baseline, 70% had bowel dysfunction and a mean quality-of-life score of 0.33. Bowel dysfunction was the only independent predictor of lower baseline quality of life. At follow-up, severe incontinence and high bowel burden predicted lower scores. Incomplete injury predicted improvement.

**Conclusion:**

Bowel dysfunction is common and closely linked to reduced quality of life after spinal cord injury. Early and sustained management is essential.

## LAY ABSTRACT

People with spinal cord injuries often struggle with bowel function, which can greatly affect their daily lives and overall well-being. We linked medical records from Sweden’s national rehabilitation registry with a community survey of 226 adults to see how bowel problems and quality of life changed over time. At first rehabilitation, 7 out of 10 participants had bowel issues and reported lower health and mobility. After several years, nearly 30% saw improvements in bowel function, but over 40% still had ongoing problems. Those with incomplete injuries were more likely to improve. We also found that severe bowel symptoms, such as incontinence and constipation, were closely tied to poorer quality of life both early on and at follow-up. These results show that bowel care is a lasting challenge for many people after spinal cord injury and highlight the importance of early, ongoing support and treatment to enhance well-being.

For individuals with spinal cord injury (SCI), loss of voluntary bowel control presents a major challenge, with substantial negative impacts on quality of life (QoL) (1–3). Neurogenic bowel dysfunction (NBD) – including faecal incontinence, constipation, and faecal impaction – is among the most prevalent and burdensome consequences of SCI (4). A recent systematic review and meta-analysis reported that approximately 75% of individuals with SCI experience some degree of bowel dysfunction, with over half presenting with moderate-to-severe symptoms (4). Constipation affects about 55% of patients, and faecal incontinence occurs in roughly 57%, and both symptoms are significantly associated with decreased QoL (3).

Even with an effective bowel routine, bowel management can take 1–2 h daily or every other day, affecting school, work, and social life, and hindering community reintegration. Prolonged bowel care, often exceeding 30 min, is independently associated with poorer QoL (4). Moreover, for many patients, loss of bowel control is more distressing than loss of ambulation (5), leading to anxiety and emotional distress (3).

Bowel dysfunction type and severity are largely determined by the neurological level and completeness of the SCI. Upper motor neurone injuries (UMN) at or above thoracic (T) 12 cause supraconal (hyperreflexic) bowel, characterized by increased colonic and sphincter tone, prolonged transit, and constipation alternating with incontinence. On the other hand, lower motor neurone injuries (LMN) below T12 result in hyporeflexic bowel, marked by reduced tone and flaccidity, leading to poor evacuation and leakage (6).

Patients with SCI generally experience lower QoL compared with the general population (7, 8). QoL is not determined by injury level alone, but is influenced by a variety of physical, cognitive, and psychosocial factors. Participation in daily activities plays a central role (9), and NBD is among the strongest physical determinants of reduced QoL in SCI (3).

Longitudinal studies show that life satisfaction and health-related QoL (HRQoL) can remain stable over time, with 1 study reporting that life satisfaction remained largely unchanged from 1 to 5 years post-injury (10). Another investigation found stable HRQoL over 6 years, with notable improvements in depressive symptoms and global QoL among individuals with more severe injuries (11). However, little is known about the progression of bowel dysfunction itself, and its long-term implications regarding QoL. Scarce longitudinal data are available describing NBD progression and its relationship with HRQoL. Understanding these patterns is essential for guiding long-term management and rehabilitation planning.

In the present study, we aimed to characterize longitudinal changes in NBD and HRQoL in a national cohort of adults with SCI, and to identify demographic and injury-related factors that predict persistent NBD burden and reduced QoL scores. By integrating registry and survey information, this study addresses gaps in SCI research related to NBD, including its long-term evolution and implications for QoL. To our knowledge, no previous study has compared NBD status at primary rehabilitation with its progression over time.

## METHODS

### Study design and data sources

We performed a combined registry-based and cross-sectional survey study, which integrated self-reported outcomes on functioning, contextual factors, lesion characteristics, and QoL from the 2024 Swedish International Spinal Cord Injury (InSCI) Community Survey (12, 13) with clinician-reported baseline data from the Swedish National Quality Registry for Rehabilitation Medicine (SveReh) (14, 15). This study design enabled longitudinal assessment of bowel dysfunction and HRQoL from discharge after primary inpatient rehabilitation to community follow-up. A pseudonymization procedure was used to securely link InSCI responses to corresponding SveReh records.

### Participants

Individuals were eligible for inclusion if they met the following international InSCI criteria: age ≥ 18 years, living in the community in Sweden (independently or with support), having a traumatic SCI (including cauda equina syndrome) or non-traumatic SCI, and being ≥ 12 months post-injury. Exclusion criteria were congenital conditions (e.g., spina bifida), neurodegenerative diseases (e.g., multiple sclerosis and amyotrophic lateral sclerosis), and peripheral nerve disorders (e.g., Guillain–Barré syndrome).

### InSCI survey (self-reported)

The InSCI questionnaire is based on the International Classification of Functioning, Disability and Health (ICF), and its structure has been described previously (16). The Swedish survey was administered, using a paper or electronic format, in spring 2024 (March–June). Standard components include the EQ-5D-5L and the Spinal Cord Injury Secondary Conditions Scale (SCI-SCS) (17). To enable detailed assessment of bowel symptoms, the Swedish version also included the Constipation Scoring System (CSS) (18) and St. Mark’s Incontinence Score (Vaizey Score) (19, 20). CSS scores range from 0–30, with a score of ≥ 15 indicating clinically significant constipation. Vaizey scores range from 0–24, and a score of ≥ 12 indicates severe faecal incontinence. Scores on the SCI-SCS bowel item range from 1–5, and were dichotomized into low (1–2) vs high burden (3–5).

### SveReh registry (clinician-reported)

SveReh is a national registry for monitoring rehabilitation outcomes in Sweden, and was established in 1997. All rehabilitation medicine units contribute data, with an estimated inclusion rate of ~90%. For this study, we used data from only 3 SCI rehabilitation units (Gothenburg, Uppsala, and Örebro), because these units had recruited the participants included in the InSCI survey. Baseline data were collected at the time of discharge from primary inpatient rehabilitation after SCI.

SveReh provides demographic and clinical data collected on admission and discharge from primary inpatient rehabilitation – including AIS grade, neurological level, aetiology, complications, and ICF-coded bowel variables (14, 15, 21). In SveReh, “bowel disorder” indicates the presence of neurogenic bowel due to SCI, and reflects non-volitional bowel control rather than symptom severity. The InSCI follow-up incorporated the CSS, Vaizey Score, and SCI-SCS bowel item to distinguish well-functioning neurogenic bowel patterns from clinically significant complications.

The EQ-5D (22) is a widely used generic measure of health status, which was developed by the EuroQol Group, an international network of researchers established in 1987 (23). The EQ-5D-3L was utilized at baseline, while the EQ-5D-5L was applied at follow-up (22, 23). EQ-5D-5L responses were converted to EQ-5D-3L index values using the van Hout crosswalk method (24), applying Danish values due to the lack of Swedish values.

### Statistical analysis

Participant characteristics were summarized using descriptive statistics. Group comparisons were performed using χ^2^ tests for categorical variables, and Mann–Whitney *U* tests for non-normal continuous and ordinal outcomes. Binary logistic regression was used to examine predictors of adverse bowel outcomes and improvement in bowel function (change in SCI-SCS). Linear regression was used to evaluate predictors of EQ-5D index values at baseline and follow-up, and predictors of change in HRQoL. Ordinal regression was used when the SCI-SCS item was analysed as an ordinal variable. Statistical significance was set at *p* < 0.05. Analyses were conducted using IBM SPSS Statistics, version 29.0.0.0 (IBM Corp, Armonk, NY, USA).

### Bias assessment

To assess selection bias, χ^2^ tests were used to compare participants with paired data available (*n* = 157) with excluded participants (*n* = 69) in terms of sex, injury level, completeness, and aetiology. Additionally, responders were compared with non-responders (*n* = 380) in terms of baseline characteristics.

## RESULTS

### Demographic characteristics

**Fig. 1:**
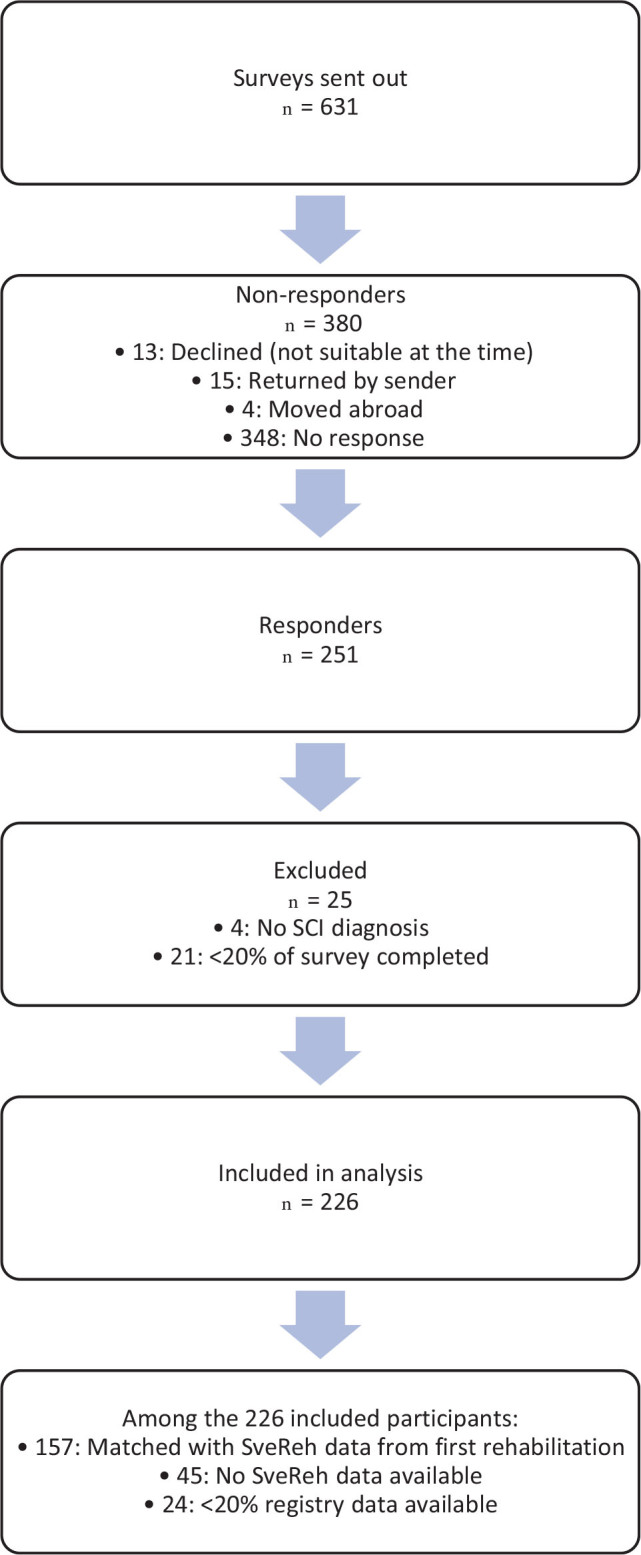
Participant inclusion.

Among the 631 individuals contacted, 251 responded to the InSCI survey, and 25 were excluded due to missing or invalid data, yielding 226 participants (Fig. 1). Table I presents their demographic characteristics. Bowel function data from both time-points were available for 155 participants, and paired EQ-5D data were available for 102 participants. A total of 157 participants (69.5%) had paired data available for at least 1 domain.

**Table I:** Demographic characteristics of the study population (*n* = 226) according to injury type

Characteristic	TSCI (*n* = 185)	NTSCI (*n* = 41)
Total *N*	185	41
*N* (%)	185 (82.0%)	41 (18.0%)
Cause: Falls	69 (37.0%)	–
Cause: Traffic	50 (27.0%)	–
Cause: Tumour	–	10 (24.4%)
Benign tumour	–	9 (22.0%)
Malignant tumour	–	1 (2.4%)
Cause: Infection	–	10 (24.4%)
Cause: Degenerative	–	9 (22.0%)
Male sex	134 (72.4%)	23 (56.1%)
Age at injury, mean (median, range, SD)	50.5 (54.5, 16–76, 18.2)	51.1 (55.0, 27–76, 13.4)
Time since injury in years, mean (median, range, SD)	8.2 (6.0, 1–58, 9.3)	8.7 (5.0, 1–45, 9.3)
Tetraplegia	93 (50.3%)	13 (31.7%)
Paraplegia	75 (40.5%)	20 (48.8%)
Complete lesion	39 (21.0%)	6 (14.6%)
Incomplete lesion	129 (69.9%)	27 (65.9%)
Full recovery	17 (9.2%)	8 (19.5%)

TSCI: traumatic spinal cord injury; NTSCI: non-traumatic spinal cord injury.

Compared with excluded individuals (*n* = 69), included participants were more often male, were older at injury, had a traumatic SCI (TSCI), and had a shorter time since injury (*p* < 0.01 for all). Neurological level and injury completeness did not significantly differ between groups (Table II), indicating a similar distribution of injury severity across groups.

**Table II:** Characteristics of participants included vs excluded in paired comparison over time

Characteristic	Included (*n* = 157)	Excluded (*n* = 69)	*p-*value	Statistical test
*N*	157	69		
TSCI, *n* (%)	136 (86.6%)	49 (71.0%)	0.009	χ^2^
NTSCI, *n* (%)	21 (13.4%)	20 (29.0%)	0.009	χ^2^
Male, *n* (%)	118 (75.2%)	39 (56.5%)	0.008	χ^2^
Age at injury, median (SD)	58 (16.5)	42.5 (16.4)	< 0.001	Mann–Whitney *U* test
Time since injury in years, median (SD)	5 (2.3)	10 (14.1)	< 0.001	Mann–Whitney *U* test
Tetraplegia	80 (51.0%)	26 (37.7%)	0.161	χ^2^
Paraplegia	62 (39.5%)	33 (47.8%)	0.161	χ^2^
Complete lesion	30 (19.1%)	15 (21.7%)	0.445	χ^2^
Incomplete lesion	112 (71.3%)	44 (63.8%)	0.445	χ^2^
Full recovery	15 (9.6%)	10 (14.5%)	0.445	χ^2^

TSCI: traumatic spinal cord injury; NTSCI: non-traumatic spinal cord injury.

### Non-responders

Compared with responders, non-responders to the InSCI survey (*n* = 380) were younger (median age 42 vs 58 years). These groups were similar in terms of gender distribution and time since injury. Approximately half of non-responders lacked information regarding neurological level and AIS; among those with available data, AIS D was most common. These findings indicate that non-responders primarily differed in age, with no strong evidence of other systematic differences.

### Baseline NBD at time of discharge from primary inpatient rehabilitation (n = 155; SveReh, clinician-reported)

At baseline, 70.3% of participants had a documented NBD, and 11.6% had incontinence (~30% were missing incontinence data). NBD was more common in TSCI than non-traumatic SCI (NTSCI) (*p* = 0.014). Completeness was strongly associated with bowel disorder; all participants with complete injuries had a recorded NBD (*p* <0.001). Analysis of level of injury revealed a non-significant trend towards a higher prevalence of NBD in tetraplegia (*p* = 0.062). In logistic regression, NTSCI remained associated with lower odds of bowel disorder (OR = 0.27, *p* = 0.022). Sex and age were not predictive. Completeness was excluded due to quasi-complete separation.

We compared patients with an upper vs lower motor neurone pattern. However, only a small sample had an LMN (*n* = 16). NBD was more common in UMN (C2–T11) than LMN (T12–S4/5) injuries (76.8% vs 56.3%, *p* = 0.017). These groups had equal rates of incontinence (both 12.5%). The AIS distributions differed, with most cases classified as AIS D (Table III). Notably, these comparisons represent only the participants with available NLI data.

**Table III:** Baseline bowel outcomes according to neurological level (UMN vs LMN)

Variable	UMN (C2–T11)	LMN (T12–S4/5)	*p*-value
*n* (%)	112 (72.3%)	16 (10.3%)	–
Bowel disorder (SveReh)	76.8%	56.3%	0.017
Bowel incontinence (SveReh)	12.5%	12.5%	0.197
AIS A	17.0%	12.5%	<0.001
AIS B	15.2%	12.5%	–
AIS C	13.4%	12.5%	–
AIS D	51.8%	62.5%	–
AIS missing	2.7%	0%	–
Traumatic aetiology (TSCI)	88.4%	93.8%	0.099
Sex (male)	76.8%	56.3%	0.193

LMN estimates should be interpreted cautiously due to the small number of cases.

UMN: injuries at or above C2–T11; LMN: injuries at or below T12–S4/5; SveReh: Swedish National Quality Registry for Rehabilitation Medicine; AIS: American Spinal Injury Association Impairment Scale; TSCI: traumatic spinal cord injury.Bowel disorder and bowel incontinence were clinician-reported in SveReh on discharge after primary inpatient rehabilitation. *p*-values were obtained from χ^2^ tests.

### Baseline EQ-5D-3L at time of discharge from primary inpatient rehabilitation (n = 102)

At baseline, the mean EQ-5D-3L index was 0.330 (SD = 0.329, range: −0.594 to 0.814). The most commonly affected dimensions were mobility (94.1%), usual activities (90.2%), and pain/discomfort (87.3%). Fewer participants reported problems with anxiety or depression (52.0% reported no issues). NBD was associated with lower mean EQ-5D scores (0.260 vs 0.519, Z = −3.36, *p* < 0.001). In multivariable regression, NBD remained the only significant predictor of EQ-5D (B = −0.247, *p* = 0.004). No other demographic or injury-related variables were associated with baseline HRQoL.

### Follow-up NBD (n = 226; InSCI survey)

At follow-up, clinically significant constipation was rare, with a CSS of > 15 reported by 3.1%. The average Vaizey score was 9.49 (SD = 5.0). Half of participants (51.3%) reported SCI-SCS ≥ 3, indicating moderate-to-severe bowel burden. Across the CSS, Vaizey, and SCI-SCS scales, we observed no associations with sex, neurological level, completeness, aetiology, or time since injury (*p* > 0.05 for all), with the exception of a higher SCI-SCS burden in participants injured <6 years ago (Z =−2.84, *p* = 0.005). In adjusted models, no injury-related variables predicted SCI-SCS severity.

*Upper vs lower motor neuron pattern.* No significant differences were observed between UMN and LMN groups for SCI-SCS burden, Vaizey ≥ 12, or CSS ≥ 16 (all *p* > 0.55). These results are presented in Table IV and reflect the subset with available NLI data.

**Table IV:** Bowel outcomes at follow-up according to neurological level (UMN vs LMN)

Outcome/Variable	UMN (Th11+)	LMN (Th12–S4/5)	*p*-value
*n* (%)	115 (52.0%)	17 (7.7%)	–
SCI-SCS ≥3	54.8%	58.8%	0.566
Vaizey ≥12	36.3%	47.1%	0.622
CSS ≥16	3.6%	5.9%	0.759

Outcomes include self-reported bowel burden (SCI-SCS), faecal incontinence severity (Vaizey score), and constipation severity (CSS).

The small LMN subgroup limits statistical power.

Categories reflect established clinical cut-offs (SCI-SCS ≥3, Vaizey ≥12, CSS ≥ 16).

UMN: injuries at or above Th11; LMN: injuries at or below Th12; SCI-SCS: Spinal Cord Injury Secondary Conditions Scale.

*p*-values were obtained from χ^2^ tests.

### Follow-up EQ-5D-5L at follow- up, InSCI survey (n = 226)

Mean EQ-5D-5L score was 0.730 (SD = 0.17). The most affected dimension was mobility: 38.5% reported being unable to walk, while only 15.9% had no mobility limitations. In the self-care domain, 48.2% reported no problems, whereas 16.4% were unable to wash or dress independently. Those with tetraplegia reported significantly lower scores than those with paraplegia (*p* < 0.001), and those injured ≥ 7 years ago reported higher EQ-5D-5L scores compared with those injured more recently (*p* = 0.029).

Higher bowel symptom burden (SCI-SCS ≥ 3), Vaizey ≥ 12, and CSS > 15 were all associated with lower EQ-5D-5L (all *p* ≤ 0.004). In multivariable models, severe incontinence and high SCI-SCS burden independently predicted lower EQ-5D-5L scores; tetraplegia remained a strong predictor.

### Change in NBD over time (n = 155)

Across timepoints, 27.7% improved, 13.5% worsened, 41.3% had persistent symptoms, and 14.8% remained stable with no reported bowel issues at either time point.

Injury completeness significantly predicted improvement: individuals with complete injuries had lower odds of bowel function improvement compared with those with incomplete injuries (OR = 0.23, *p* = 0.002). No associations were seen with sex, level, aetiology, or time since injury.

Comparison of patients with an upper vs lower motor neurone pattern revealed that improvement was more common in UMN injuries (37.0% vs 6.3%, *p* = 0.001). The vast majority of LMN cases exhibited no improvement (93.8%) (Table V).

**Table V:** Change in bowel function over time according to neurological level (UMN vs LMN)

Outcome	UMN (C2–T11)	LMN (T12–S4/5)	*p*-value
*n* (%)	108 (71.5%)	16 (10.6%)	–
Improved bowel function	37.0%	6.3%	0.001
No improvement/same or worse	63.0%	93.8%	–

Change in bowel function reflects improvement vs no improvement/worsening based on paired SveReh and InSCI data (clinician-reported ICF bowel dysfunction on rehabilitation discharge and the SCI-SCS bowel item at follow-up).

The LMN subgroup is small (*n*=16), and results should be interpreted cautiously.

UMN: injuries at or above C2–T11; LMN: injuries at or below T12–S4/5.

*p*-value was obtained from χ^2^ test.

### EQ-5D change over time (n = 102)

EQ-5D significantly increased from baseline to follow-up (Z = 3.43, *p* < 0.001), with improvement observed in two-thirds of participants. Linear regression models using both dichotomized and continuous bowel symptom measures (SCI-SCS, Vaizey, and CSS) did not identify NBD as a significant predictor of change in EQ-5D index scores from baseline to follow-up. When bowel symptoms were entered as binary variables (e.g., high vs low burden), no significant association with EQ-5D improvement was observed. Similarly, when bowel symptom scores were included as continuous or ordinal predictors, change in EQ-5D was not significantly associated with any of the bowel measures: SCI-SCS (*p* = 0.594), Vaizey (*p* = 0.156), or CSS (*p* = 0.834). These findings suggest that, although NBD cross-sectionally affects quality of life, it did not independently predict change in EQ-5D over time in this sample.

## DISCUSSION

This study provides new longitudinal evidence regarding NBD and QoL among adults living with SCI. We linked clinician-reported data from primary rehabilitation with self-reported outcomes collected several years later, enabling us to track the persistence and progression of bowel symptoms over time. On discharge after primary rehabilitation, approximately 70% of participants had clinically documented NBD, and reported substantially reduced QoL. At follow-up, nearly 30% had experienced improvements in bowel function; however, more than 40% reported persistent symptoms. Individuals with incomplete injuries were more likely to experience improvement, while those with complete or cervical injuries had a higher risk of long-term bowel burden. Across both time-points, more severe bowel symptoms – including constipation, faecal incontinence, and high SCI-SCS scores – were consistently associated with poorer QoL.

Taken together, these findings highlight that NBD remains a significant and enduring challenge after SCI, having clear implications regarding well-being and daily functioning. The longitudinal design further demonstrated that although individuals with greater bowel burden reported lower QoL at both baseline and follow-up, the presence of NBD did not predict the degree of QoL improvement over time. This suggests that individuals with persisting bowel-related challenges may adapt to their circumstances and experience general improvements in perceived health.

With this overall pattern established, it is important to consider the potential sources of bias and the representativeness of the sample. The analysed paired sample differed from the 69 excluded participants in several characteristics – including sex, injury aetiology, age, and time since injury – but the groups did not differ significantly in neurological level (tetraplegia vs paraplegia) or degree of impairment (complete vs incomplete). This suggests that, despite some demographic selection bias, the overall range of neurological severity was preserved, supporting that the findings related to bowel function and HRQoL can be generalized across SCI populations.

The older age and shorter time since injury in the analytic sample reflect both clinical and registry-related patterns. The longer time since injury among excluded individuals likely reflects the previously limited availability of electronic registry data. SveReh (formerly WebRehab) was launched in 1997, but was only electronically implemented from 2007 onwards; therefore, the registry may not contain rehabilitation data for individuals injured before 2007 (14). Additionally, the inclusion of more recently injured and older participants aligns with current epidemiological trends showing increasing occurrence of SCI among older adults (25).

Among the 380 non-responders to the InSCI survey, SveReh data indicated that younger individuals were less likely to respond, suggesting a potential age-related participation bias. On the other hand, responders and non-responders were similar in terms of sex and time since injury. However, a large proportion of non-responders were missing data regarding injury severity, limiting conclusions regarding the influence of neurological factors on response rates. These findings underscore a persistent challenge in SCI survey research, namely that younger and potentially less-engaged individuals may be underrepresented in long-term follow-up studies. This highlights the need for targeted strategies to improve response rates and ensure broader representation over time.

The present results confirm the substantial burden of NBD following SCI, and its consistent association with reduced health-related quality of life. At the time of first rehabilitation, NBD was documented in 70.3% of participants, and all individuals with complete injuries had a recorded bowel disorder. NBD was more common in participants with TSCI than NTSCI, and we observed a trend towards higher prevalence in tetraplegia. These findings align with previous research (3, 4, 26).

Health-related quality of life at baseline, measured by EQ-5D-3L, was notably low (mean=0.33), particularly in the domains of mobility, usual activities, and pain/discomfort. In multivariable analysis, NBD was the only independent predictor of EQ-5D-3L scores, and was associated with a clinically meaningful reduction of approximately 0.25 points. At follow-up, using EQ-5D-5L (converted to 3L index values), a consistent pattern emerged: in unadjusted analyses, greater bowel symptom burden (measured by SCI-SCS, Vaizey, and CSS) was significantly associated with lower EQ-5D scores. In adjusted models, both severe incontinence (Vaizey ≥12) and high SCI-SCS burden (3–5) independently predicted lower EQ-5D-5L scores. Additionally, tetraplegia emerged as a consistent predictor of reduced HRQoL in both models, while completeness showed a weaker, but still meaningful, association. Time since injury, sex, and aetiology did not show significant associations with HRQoL in multivariable models.

The significant improvement of EQ-5D-3L scores from baseline to follow-up indicates that, on average, participants experienced gains in perceived quality of life. This was supported by a Wilcoxon signed-rank test. However, regression models showed that although NBD was predictive of cross-sectional EQ-5D, it did not predict the magnitude of change over time. This suggests that while individuals with NBD start off experiencing lower HRQoL, they improve at similar rates to those without NBD. Notably, lower EQ-5D scores persisted among participants with high bowel burden, even without further decline, emphasizing the lasting impact of bowel symptoms.

Significant constipation (CSS ≥16) was associated with lower EQ-5D in unadjusted analysis; however, the low proportion of affected participants (3.1%) limited its interpretability and inclusion in regression models. Similarly, time since injury was associated with lower SCI-SCS scores in unadjusted analysis, suggesting potential symptom improvement over time, but this was not confirmed in adjusted models.

The majority of participants with bowel symptoms reported that their symptoms remained stable or worsened over time; 41.3% had persistent symptoms, while only 27.7% of participants reported improvement. Completeness of injury was significantly associated with bowel function change, with improvement being more likely among individuals with incomplete injuries. In logistic regression, completeness remained the only significant predictor of improvement, as no associations were observed with sex, level, aetiology, or time since injury.

Analysis of the Vaizey score revealed that female sex was independently associated with higher incontinence scores. However, the overall model explained little variance, and was not statistically significant. The lack of associations with injury characteristics suggests that bowel symptoms are likely shaped by factors not captured in this study, such as autonomic function, management routines, and psychosocial influences.

Across baseline, follow-up, and longitudinal analyses, we examined whether disproportionately greater bowel dysfunction was experienced by individuals with LMN injuries, which are typically associated with a reflexic bowel and greater management challenges. Notably, LMN injuries were relatively uncommon in this cohort. NBD on discharge and bowel symptom burden at follow-up did not differ significantly between the LMN and UMN groups; however, participants with LMN injuries were markedly less likely to show improvement over time. These findings must be interpreted cautiously given the small LMN sample, and the absence of LMN-specific bowel management variables in SveReh and InSCI. Nonetheless, the pattern aligns with the clinical experience that LMN bowel dysfunction presents persistent challenges and limited recovery potential.

Recent studies (4, 26, 27) have confirmed that bowel symptoms negatively impact quality of life after spinal cord injury, and the present investigation adds important longitudinal evidence by assessing both NBD and HRQoL at 2 distinct time-points. This dual-time-point design promotes a more comprehensive understanding of the persistence and progression of bowel-related burden over time, and of its sustained influence on health-related quality of life. Collectively, the present findings confirm that NBD is a robust and persistent issue in SCI, with consistent cross-sectional impacts on HRQoL and a clear association with neurological severity. However, NBD was not a significant predictor of HRQoL improvement over time, nor did it explain longitudinal changes in functioning. This underscores the complexity of bowel outcomes after SCI, and highlights the importance of early and sustained bowel management across the rehabilitation continuum.

### Limitations

This study has several limitations. First, although the InSCI survey reached a broad population, the response rate was modest (251 of 631; 40%), and only 157 participants (69.5%) had paired data available from both the InSCI survey and the SveReh registry. Participants with paired data available were more likely to be male, to be older at injury, to have a TSCI, and to be further post-injury, which introduces potential selection bias and limits the generalizability of these findings to younger individuals, persons with NTSCI, and those early in recovery. However, we found no significant differences in neurological level or injury completeness, mitigating concerns about representativeness relating to injury severity.

Second, although all utilized assessment tools (CSS, Vaizey, SCI-SCS, and EQ-5D) are validated, the combination of registry-based clinical data and self-reported survey data collected at different time-points introduces methodological variability. The results may have been influenced by changes in clinical practice, recall bias, and evolving symptom perception. Additionally, all analyses were based on complete cases; no imputation was performed.

### Conclusion

This study highlights the persistent burden of bowel dysfunction among individuals with spinal cord injury, and its consistent association with reduced health-related quality of life across the rehabilitation continuum. Bowel dysfunction was highly prevalent at first rehabilitation, and was independently associated with lower EQ-5D scores both cross-sectionally and at follow-up. Although participants with bowel dysfunction reported lower HRQoL at both time-points, bowel symptoms did not predict the degree of improvement over time. Instead, neurological completeness emerged as the most consistent predictor of both HRQoL gains and bowel function improvement. These findings underscore the need for early identification, targeted intervention, and long-term follow-up strategies to manage bowel dysfunction and improve outcomes in SCI rehabilitation.

## Data Availability

Data are available upon reasonable request.
